# Seasonality as a risk factor for deaths in Parkinson's disease

**DOI:** 10.1016/j.clinsp.2024.100506

**Published:** 2024-10-25

**Authors:** Marcelo C.M. Fonseca, Dayan Sansone, Daniela Farah, Ana Claudia Fiorini, Carla A. Scorza, Fulvio A. Scorza

**Affiliations:** a Departamento de Ginecologia, Escola Paulista de Medicina/Universidade Federal de São Paulo (EPM/UNIFESP), São Paulo, SP, Brasil; b Departamento de Fonoaudiologia, Escola Paulista de Medicina/Universidade Federal de São Paulo (EPM/UNIFESP). São Paulo, SP, Brasil; c Programa de Estudos Pós-Graduado em Fonoaudiologia, Pontifícia Universidade Católica de São Paulo (PUC-SP), São Paulo, SP, Brasil; d Disciplina de Neurociência, Escola Paulista de Medicina/Universidade Federal de São Paulo (EPM/UNIFESP). São Paulo, SP, Brasil

**Keywords:** Parkinson disease, Circadian rhythm, Mortality, Brazil

## Abstract

**Background:**

According to growing evidence, sleep disruption harms biological processes and circadian homeostasis. Diurnal motor symptom volatility in Parkinson's Disease (PD) has been extensively studied. Few studies examined seasonal variability in PD symptoms, some showing it and others not.

**Objective:**

To investigate whether PD patients' deaths follow a rhythmic pattern due to circadian rhythm alterations. Methods: This study used only unidentified patient databases. People with PD, ICD10 code G20, in at least one death certificate field were selected. The Continuous Wavelet Transform and Fourier Transform were checked for oscillation and its duration.

**Results:**

The 18-year analysis found 43,072 PD deaths. The Continuous Wavelet transform revealed a 351.87-day annual component (p < 0.05). Winter in the southern hemisphere saw more deaths, mainly in July. The Continuous Wavelet transform identified a significant daily component (p < 0.05) of 22.81 hours. Fatalities peaked around 9 a.m. Pneumonia is the leading cause of death in PD, and women and men have the same rhythm pattern.

**Conclusion:**

Parkinson's disease mortality in Brazil follows a pattern. Using over 40.000 death certificates from 18 years, the authors found that Parkinson's patient fatalities rise in winter and peak in July at about 9 a.m. Sunlight reduction increases mortality risk in the long term. Low sunshine lowers temperatures, increasing short-term death risk. This is crucial because it prioritizes the sun, seasons, and circadian rhythm over low temperatures.

## Highlights

• Understanding the impact of circadian rhythms on Parkinson's disease pathogenesis and mortality can revolutionize healthcare.
• The authors investigated whether the deaths of Parkinson's disease patients follow a rhythmic pattern due to variations in the circadian rhythm.
• During winter was seen more deaths, and fatalities peaked around 9 a.m.
• Sunlight reduction increases mortality risk in the long term in patients with Parkinson's disease.

## Introduction

At all levels of organization, from gene expression to intra- and inter-organ physiological coordination, the circadian organization of biological processes plays a fundamental role. [1,2]

Primary neurodegenerative processes affect several brain regions, especially those involved in sleep regulation, alertness, and circadian rhythms, causing interruption of sleep-wake cycles and circadian dysregulation. [3,4]

Increasing evidence shows the harmful effects of sleep disruption and circadian homeostasis on biological processes. There are indications that these effects are the basis of neurodegeneration. [5]

Studies indicate the association of sleep and circadian rhythm disorders with comorbidities frequently present in Parkinson's Disease (PD), such as cognitive decline, memory impairment, and neurodegeneration. [6] In fact, sleep and circadian rhythm alterations exist in most patients with an evident clinical manifestation of PD.

Diurnal volatility in PD has long been studied, particularly motor symptoms. There is a decline in overall daytime activity but an apparent fall in morning activity. [7,8] Likewise, non-motor symptoms vary throughout the day. In terms of autonomic function, there is a disruption in the circadian rhythm of blood pressure and heart rate control. [9,10] There is a wide range of impairments of the sleep-wake cycle, including problems with nocturnal sleep and daytime wakefulness, circadian system dysfunction, alteration of visual performance with worsening contrast sensitivity in the early afternoon, and fluctuation in response to dopaminergic treatments with worsening motor symptoms in the afternoon and at night. [11, 12, 13, 14]

Only a few studies have looked into the possibility of seasonal variability in PD symptoms, some demonstrating it and others not. However, consistent with patients' and health professionals' subjective perceptions, the symptoms appear less intense in the summer when the nights are shorter and more pronounced in the winter, although robust evidence of seasonal fluctuations in PD symptoms is lacking.

Postuma et al. showed no difference in UPDR motor scores when comparing the four seasons, and Goetz et al. found no seasonality in hallucinations. [15]

Wamelen et al., on the other hand, found substantial seasonal differences in cardiovascular, sensory alterations, falls, and hallucinations in PD patients, with winter symptoms worsening compared to summer. They also noticed a worsening smell during the winter and spring. [16] Recently, Wang et al. observed that Chinese Parkinson's patients exhibit less severe autonomic dysfunction and REM Sleep Behavioral Disorder symptoms in the summer than in the winter. Furthermore, they reported fewer sleep disruptions in the summer than in the spring and winter. [17]

These fluctuations in the clinical manifestations of PD can be explained by the pathology of the SCN, which results in a shift in circannual regulation in PD. The magnitude of sleep and circadian dysfunction in PD and its influence on the pathophysiology of PD is still unclear.

There are seasons of the year when deaths are more prevalent. Deaths from various causes have an annual cycle, peaking during the winter and decreasing in the summer. [18] Therefore, the authors decided to investigate whether these circadian rhythm alterations in PD could cause a rhythmic pattern in the deaths of patients affected by PD.

## Methods

This study only used databases of patients who were not identified. Therefore, according to the institution's rules, the authors did not need to submit this study to the institutional review board.

Data on PD deaths were taken from the Mortality Information System (SIM) database of the Department of Informatics of the Unified Health System (DATASUS). [19] The years sampled were from 2000 to 2017.

People with PD, ICD10 code G20, in at least one of the possible fields to fill in the death certificate (line A, line B, line C, line D, line II, and underlying cause) were selected from the database. The death was counted for analysis if there was a PD code in these fields.

All extraction analyses and graphics were performed with the statistical programming language R [20] and Python language. [21]

To analyze whether there is a rhythm in Parkinson's deaths, the method by Damineli et al. was used. [22] Transforming the recorded dates of deaths into Julian days is the basis of this method to obtain a linear time series of deaths involving PD. To avoid masking the rhythms, trends (larger cycles) and noise (smaller cycles) were isolated in this time series.

The analysis to verify if there was and the period of oscillation was carried out by the Continuous Wavelet Transform and the Fourier Transform. The authors used a filter of a minimum period of 4 and a maximum of 1500 days for supra-daily rhythms. For daily or infra-daily rhythms, the authors applied a filter with a minimum period of 4 and a maximum of 40 hours.

To analyze the existence of a daily rhythm, the time of death has to be recorded. This recording became part of the SIM-DATASUS database from 2006 onwards. Thus, the authors had six years less for the annual rhythms to investigate and analyze the circadian rhythm.

## Results

There were 43,072 deaths involving PD in the 18 years of analysis, an average of 2,392.8 per year. There was constant growth over the years sampled. In the first year, there were 1,061 deaths involving PD; in 2017, there were 3,759. In total, men accounted for 55% of the deaths.

**Fig. 1:**
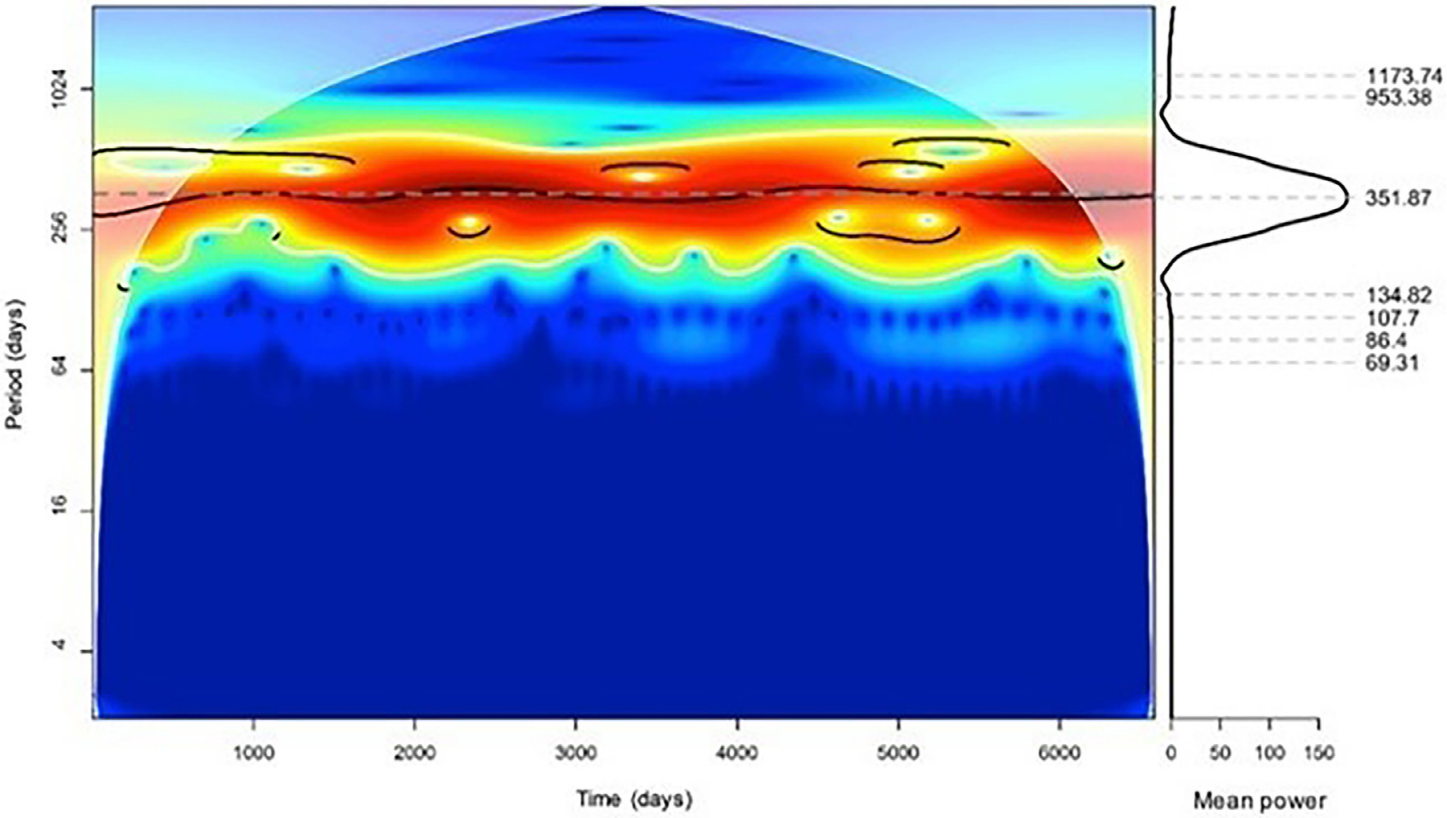
Continuous wavelet transform.

The Continuous Wavelet transform showed a significant annual component (p < 0.05) with a period of 351.87 days (Fig. 1). The application of the Fourier transform showed an annual component recorded in 365.9 days (Supplementary Material 1).

The distribution of deaths over the months was investigated by demonstrating an annual rhythm. There were more deaths in the winter (in the southern hemisphere), with most deaths occurring in July (Supplementary Material 2).

**Fig. 2:**
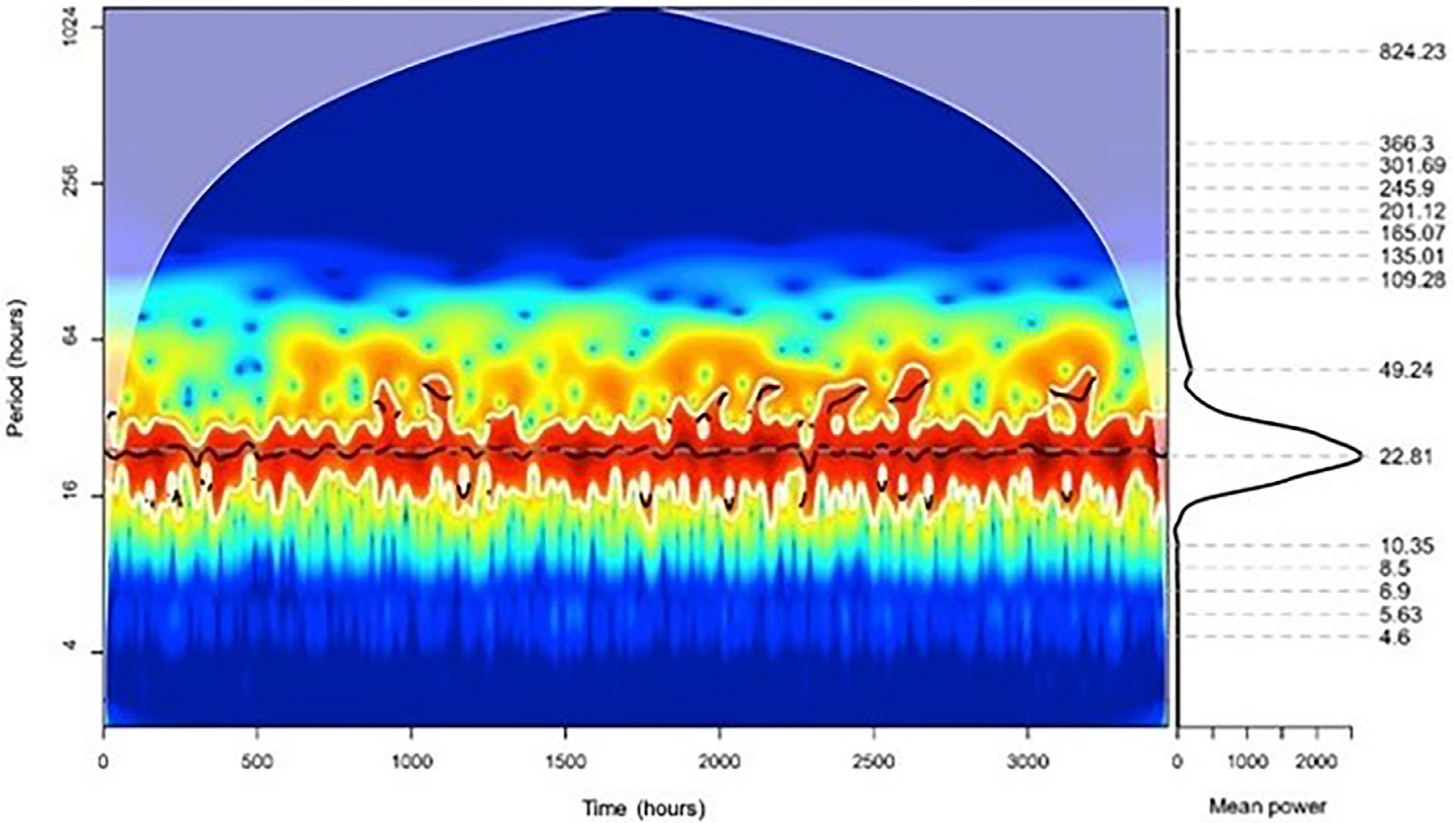
Continuous wavelet transform.

To analyze the daily rhythms, the Continuous Wavelet transform in the time series showed a significant daily component (p < 0.05) with 22.81 hours (Fig. 2). The Fourier transform peaked in the daily component (24 hours) (Supplementary Material 3).

**Fig. 3:**
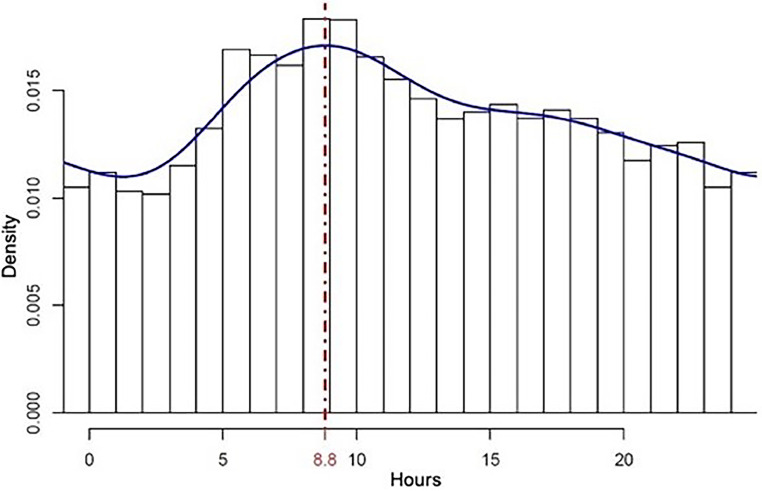
Distribution of deaths along the day.

With the daily rhythm confirmed, the distribution of deaths over the hours of the day was investigated. There was a greater concentration of fatalities in the first morning hours, with the density peak around 9 a.m. (Fig. 3).

### Women and men

Women and men exhibit the same rhythm pattern as the whole population. Winter is the deadliest season for both populations. The peak of deaths occurs between 8 and 9 a.m. in both populations, with no difference between the hours of death in men and women (p = 0.35). Women with PD die older than men (p < 0.000). Although the numbers differ, men and women die from the exact nine causes (p < 0.000). Death certificates revealed other gender differences (Table 1).

**Table 1:** Men and women characteristics retrieved from death certificates.

	Men (23,581)	Women (19,486)	Total (43,067)
**Mean Age**^a^	78.5	81.1	
**Education**^a^			
None^b^	10.7%	14.2%	12.3%
Incomplete elementary school	24.5%	25.3%	24.8%
Complete elementary school	17.6%	17.2%	17.4%
Middle school	11.4%	11.1%	11.3%
Complete high school^b^	10.7%	6.1%	8.6%
Empty, ignored, not reported^b^	25.2%	26.1%	25.6%
**Race**^a^			
White^c^	74.1%	78.4%	76.0%
Black^c^	3.1%	2.3%	2.8%
Yellow	0.9%	0.9%	0.9%
Brown^c^	16.8%	13.3%	15.2%
Indigenous	0.1%	0.0%	0.1%
Empty, not informed	5.0%	5.1%	5.0%
**Top-3 *causa mortis* (ICD code)**^a^,^e^			
J189^d^	19.4%	17.3%	18.4%
J180*	9.0%	8.6%	8.9%
J690^d^	7.1%	6.3%	6.7%

a Chi-Squared test p < 0.000.

b,c,d The adjusted residuals analysis showed significant differences (p < 0.05).

e Since there were approximately a thousand different causes of death, the authors opted to show the three leading causes of death in the table.

## Discussion

To the best of our knowledge, this is the first demonstration of a rhythm in Parkinson's disease-related deaths. The authors found that deaths of Parkinson's patients in the southern hemisphere increase during winter, when temperatures are typically lower, peaking in July and around nine o'clock in the morning. This demonstration used real-world data from over forty thousand death certificates over 18 years.

Yet, epidemiological research on the association between temperature and mortality risk is hampered by a wide range of optimal temperatures, which is associated with the lowest risk of mortality (between 19°C and 30°C), the different periods of lag between exposure to cold and mortality, which can be as long as 3 or 4 weeks, [23,24] causing the majority of deaths to occur on days with moderately cold temperatures and the heterogeneity of this association across populations as a result of adaptive responses, acclimatization, and variability in susceptibility factors. [25, 26, 27]

Moreover, given that winters in tropical southern hemisphere countries, such as Brazil, are significantly milder than those in the northern hemisphere, the authors were prompted to try to explain these findings on Parkinson's patients in a way that the explanation hypotheses go beyond those currently available, which involve temperature as the primary exposure, increased risk of mortality as the outcome and the mediation, the process through which an exposure causes disease, implies the Virchow triad and/or infections (Supplementary Material 4).

The plane perpendicular to the earth's spin axis is tilted in relation to the plane of the sun's path in the sky. These two variables, the inclination of the terrestrial axis and translational movement, generate the seasons due to variations in the insolation of the planet's surface. The hemisphere tilted toward the sun has more direct beams of sunshine; therefore, more solar radiation, more energy per unit area per day, warmer temperatures, and longer daylight hours; summer days are longer than winter days. Hence, a season is a division of the year, and the quantity of daylight hours is the key metric used to characterize seasons. [28]

Light is the most significant and powerful synchronizer for the human circadian system. [29,30] Body rhythms are mainly controlled by the SCN, the body's master clock. They are reconciled with the 24 hours of the day by light and the hormones it influences, such as melatonin.

According to epidemiological studies, high-light exposure is associated with a lower risk of PD. Two studies found that higher latitudes, where yearly light exposure is much lower, have a higher incidence of PD (up to 56%). [31,32] The activation of the SCN is hypothesized to be one of the mechanisms behind the effects of bright illuminance on mood, sleep, and circadian rhythms. [33]

Given the preceding paragraphs, the authors can hypothesize that light exposure is the primary exposure and that low temperature is a mediator. It should be noted that the mediator is both an effect of the exposure and a cause of the outcome. When a mediator is postulated, the overall effect can be split into two components, direct and indirect. The direct pathway (c’) refers to the impact of exposure on the outcome in the mediator's presence, adjusted for the mediator. The indirect effect (aMb) is the impact of exposure on the outcome via the mediator (Supplementary Material 5).

As previously indicated, the primary explanation for the increase in winter deaths involves low temperatures, as demonstrated in Supplementary Material 4. However, it appears insufficient as it does not help explain the wide range of temperatures presenting lower mortality risk, the various times of lag between cold exposure and mortality, and the demographic heterogeneity of the temperature/mortality association. As a result, the authors will emphasize the direct exposure-outcome relationship, low light exposure, and increased risk of mortality, which almost certainly contributes to the increased winter mortality in Parkinson's patients and has yet to receive much attention in the medical literature.

**Fig. 4:**
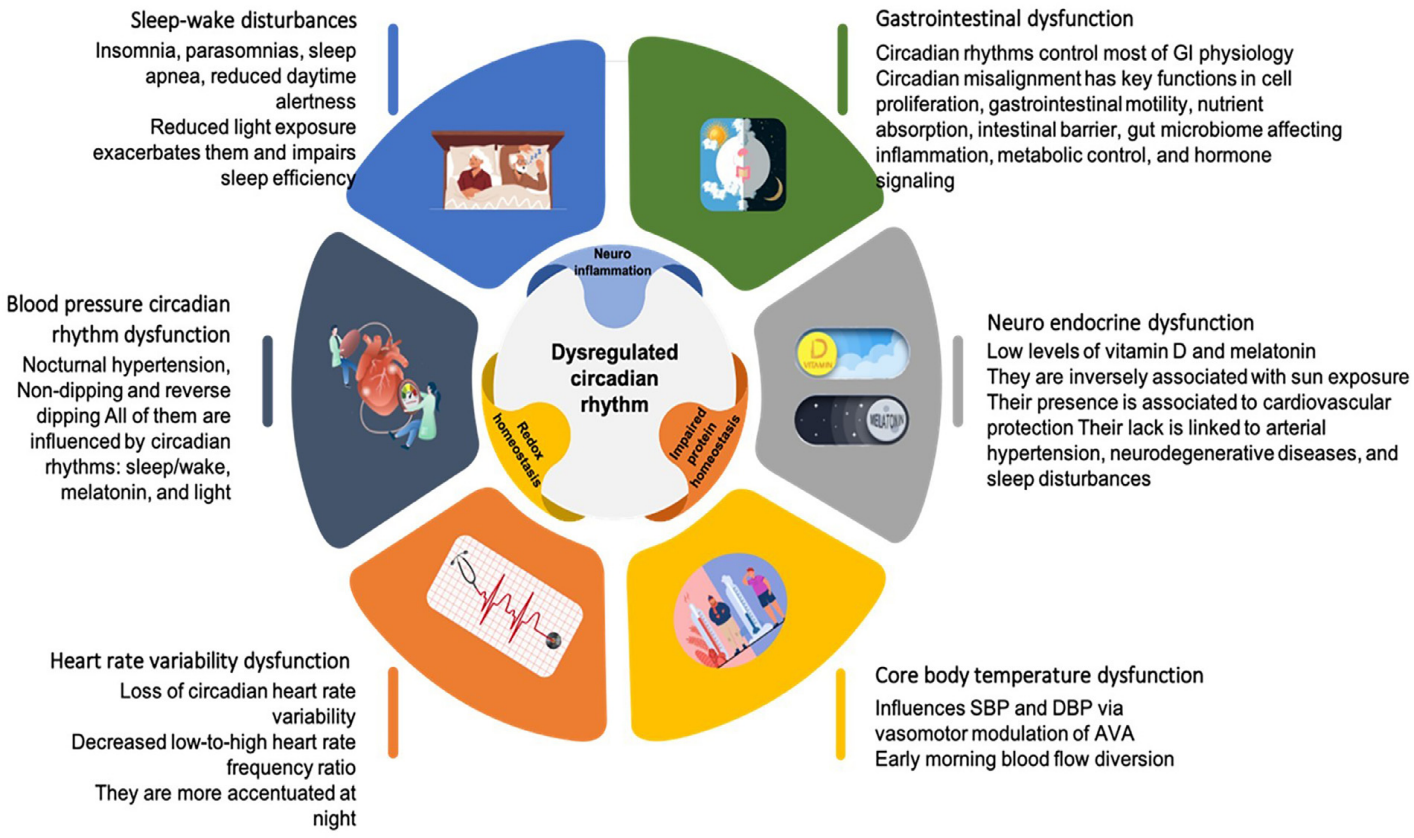
Circadian rhythm dysregulation and its repercussions on different systems.

While temperature and other environmental elements can synchronize circadian rhythms in some species, [34] in mammals, external temperature cycles are relatively poor entraining factors, [35] and daylight is the most potent external cue in humans. [36, 37, 38] However, PD patients get fewer external time cues due to decreased visual acuity and motor skills. The accumulation of alpha-synuclein in the SCN impairs the intrinsic circadian clock outputs. Hence, the disturbed rhythm of clock genes in SCN hampers its regulation of other central and peripheral clocks, which may affect behavioral and physiological processes. [39] Emerging data suggest that circadian rhythm disturbance is the primary cause of the non-motor symptoms of PD and may also alter neurodegenerative biology (Fig. 4). [39, 40, 41]

One of the most stable circadian system outputs is the sleep-wake cycle. [11] Sleep-wake disturbances are PD patients' most prevalent non-motor symptom, affecting up to 80%. [3,42] Sleep disorders have unusual symptoms when PD is present. Parkinson's disease can cause insomnia, parasomnias (REM Sleep Behavioral Disorder), sleep-disordered breathing such as sleep apnea, reduced daytime alertness, and circadian clock malfunction, which disrupts the sleep-wake balance. [11] Reduced light exposure impairs sleep efficiency and exacerbates PD patients' already prevalent sleep problems. [17]

Sleep disturbances impact the cardiovascular system, particularly blood pressure, and this adds to the fact that patients with PD usually show unique changes in their BP circadian rhythm. [43, 44, 45] One of these is nocturnal hypertension. [43] There is evidence that seasonality, rather than temperature, affects mean nocturnal SBP, with the number of daylight hours being a favorable predictor. [40] In fact, the prevalence of hypertension rises throughout the winter season, [46, 47, 48, 49] and low temperatures exacerbate hypertension [47,50, 51, 52, 53, 54, 55] and precipitate cardiovascular events such as stroke, acute myocardial infarction, and heart failure. [56, 57, 58, 59, 60, 61, 62] Parkinson's patients have a more significant proportion of non-dipping and reverse dipping than people without the condition. [44,63, 64, 65, 66] In hypertensive and some normotensive patients, abnormally low relative BP reduction during sleep (non-dipping and reverse dipping) increases the risk of fatal and non-fatal ischemic, thrombotic, hemorrhagic, and arrhythmic cardiovascular events. [67] It is noteworthy that many endogenous circadian rhythms, including sleep/wake, melatonin, and nictemeral cycles of light, noise, and ambient temperature, influence the regular dipping pattern of BP. [67,68] In PD patients, electrocardiography shows loss of circadian heart rate variability, which is more accentuated at night and decreased low-to-high heart rate frequency ratio. [16,69]

The double product (HR × SBP) is a proxy indicator of myocardial tissue oxygen demand and left ventricular workload. As its components, it has a circadian cycle. [70] It is lowered to 44% and 50% in young women and men during regular sleep compared to awake. However, the presented data suggest that it is enhanced in Parkinson's patients due to nocturnal hypertension, non-dipping and reverse dipping, and a relatively rapid heart rate reflecting the loss of sympathetic and parasympathetic balance. As a result, senior PD patients have increased left ventricular workload and myocardial tissue oxygen demand, especially during the winter with its longer nights, and this is exacerbated when the temperature drops. [70]

Since sunlight promotes vitamin D synthesis while darkness triggers melanin production, vitamin D and melatonin biosynthetic pathways are inversely associated with sun exposure. In the course of aging, vitamin D consumption and cutaneous production, as well as endogenous melatonin synthesis, are significantly reduced, resulting in a state that leads to an increase in oxidative stress, inflammation, and mitochondrial dysfunction. A lack of these molecules has been linked to the development of cardiovascular illnesses such as arterial hypertension, neurodegenerative diseases, and sleep disturbances. [47,71] It should be noted that circulating melatonin levels in PD patients are significantly lower than in age-matched healthy controls, [3,72,73] and vitamin D synthesis declines substantially during the winter. [47,71] Lower melatonin levels have been significantly associated with higher nocturnal BPs and atherosclerotic markers, indicators of cardiovascular disease incidence. [74]

In contrast, low vitamin D levels activate the renin-angiotensin-aldosterone system, which leads to a hypertensive response. [47] It is worth noting that there is substantial evidence that melatonin, an anti-inflammatory and antioxidant agent, protects against cardiac ischemia/reperfusion injury. Melatonin-treated patients exhibit a higher left ventricular ejection fraction and lower troponin levels than control persons. Patients with PD are unlikely to benefit from this cardioprotective effect due to their low melatonin content. [75] Clinical research has largely associated vitamin D insufficiency with cardiovascular disorders such as hypertension, ischemic heart disease, and heart failure; however, most of these studies are observational, making this evidence less persuasive. [76]

The authors noted that the SCN regulates the body's internal biological processes and alertness levels, which determine the daily sleep rhythm, primarily through melatonin and the Core Body Temperature (CBT) 24-hour rhythm. [70,77] The circadian rhythm of Core Body Temperature (CBT) influences SBP and DBP patterns throughout the day via vasomotor modulation of Arteriovenous Anastomoses (AVA). [70] The thermoregulatory processes encompass a significant transfer of cardiac output from the systemic circulation to the AVA, ranging from 25% to 50%. Thus, even though the CBT difference between day and night is significantly reduced in PD patients, [40] just before and upon awakening, in the early hours of the morning, there is a diversion of blood flow from the periphery to the central areas, as well as an increase in peripheral vascular resistance. The combination of increased core volume and peripheral vascular resistance causes ventricular overload, which can result in adverse consequences in a relatively brief period. [70]

Circadian rhythms drive a significant part of gastrointestinal physiology; sleep disruptions and circadian misalignment have critical roles in gastrointestinal function, inflammation, metabolic regulation, and hormone signaling. [78] Much of gastrointestinal physiology is subject to regular diurnal fluctuations, and disturbance of circadian rhythms may be a risk factor for different gastrointestinal disorders and diseases, particularly those characterized by dysmotility, such as PD. [79]

Circadian rhythms regulate cell cycle control. [79] Diurnal changes in GI motility have been documented in the human GI tract, including the stomach, small intestine, colon, and rectum. [80] Absorbing proteins, carbohydrates, lipids, and electrolytes demonstrates a circadian rhythm. Diurnal changes in intestinal barrier integrity are caused by circadian control of protein expression or trafficking in tight junctions between membrane and intracellular compartments. [81] Circadian rhythm disruption impacts the gut microbiota, leading to dysbiosis. Circadian clock gene expression is altered in GERD patients' esophageal tissue, and these changes are strongly associated with GERD severity. [82] Sleep-wake balance disruption appears to impact gastric reflux, leading to unusual exposure to esophageal acid, most likely through boosting ghrelin and decreasing leptin concentrations, increasing binge eating and food consumption. [83] In summary, given the prevalence of sleep-wake disorders in PD patients and their cascading effects in other systems, dysfunction of the gastrointestinal system will likely include components deriving from circadian rhythm misalignment.

Because PD is understood to be a multi-system condition in which inflammation, particularly neuroinflammation, plays a relevant role, the immune system in senescence cannot be overlooked. [84]

A fusion of genetic, environmental, and aging immune system elements can enable the onset and progression of PD. [84] Aging-related alterations comprise Immunosenescence, inflammageing, and an adaptive immune system with T and B cells with receptors’ reduced diversity and sensitivity to stimuli, as well as a decreased overall number of these cells and accumulation of memory cells. [85, 86, 87] The aging immune system results in high vulnerability to infections, less effective responses to vaccination, and more cases of cancer, autoimmune, and inflammatory conditions. [88] Immunosenescence is the term used to describe age-related changes in immune responses characterized by age-acquired immunodeficiency and inflammageing. The last is a low-grade, chronic inflammatory state in older people due to chronically stimulated innate and adaptive immune cells. Innate and adaptive immune systems are compromised with age and are significantly affected in several age-related disorders, such as Parkinson's and Alzheimer's. [89,90]

Considering all the described repercussions of the chronodisruption, the authors can infer that the lower exposure to light added to the PD (the direct exposure-outcome relationship) plays a dominant role in the rhythmicity of PD mortality.

Reduced exposure to light appears to be the factor whose long-term action raises the risk of mortality, whereas cold temperatures seem to be an acute factor with short-term consequences. [28] Actually, environmental temperature is a poor central circadian rhythm-synchronizing agent in mammals, acting primarily as an entraining agent of peripheral oscillators. [91] As a result, contrary to commonly held assumptions, populations living in warmer climates, such as the Brazilian population, have higher winter mortality rates, as they may experience more severe physiological consequences due to being less adapted. [92]

In line with other studies on mortality in PD, pneumonia was the leading cause of death. [93, 94, 95] This demonstrates the relevance of chronic disturbances to the circadian rhythm, which influences the physiological balance of Parkinsonians daily, particularly in the immunological, gastrointestinal, and cardiovascular systems, reducing their ability to respond to an infection. These alterations are supplemented by those caused by age, as already mentioned. PD patients experience a higher incidence of pneumonia than the general population as respiratory muscle rigidity, weakness, compromised cough reflex, and dysphagia increase the risk of PD patients to pneumonia. [96, 97, 98]

The high incidence of diagnoses that provide poor information about the underlying cause of death, dubbed “Garbage Codes” (GCs), [99] is a prominent issue of the causes of death registry's data quality. The ICD codes J189 and J180 are considered level four (lightest level) GCs. [100]

In Brazil, there was a steep decline in major GCs (Levels 1 and 2) in practically all states, indicating the government's success in improving cause-of-death statistics in Brazil during the last 20 years. [100] In a 1 to 5-star categorization, Brazil's score increased from 68.9 in 1995‒1999 to 82.5 in 2010‒2017, a 4-star country. [101]

In Brazil, older people have the most significant proportion of GCs, mainly owing to the lightest level four GCs. This occurred most likely due to their more substantial number of comorbidities, making it challenging to provide information on the death certificate of the underlying cause of death. [102, 103, 104] Of particular interest, most likely due to the documented rise in age-specific death rates from certain lower respiratory tract infections in the population aged 70 years or older, [105] the proportions of men and women with nonspecific pneumonia, a level 4GC, rose in 2016 in Brazil.

In conclusion, in Brazil, the mortality of Parkinson's disease patients follows a pattern. The authors observed that fatalities of Parkinson's patients increase during the winter, peaking in July at about nine o'clock in the morning, utilizing real-world data from over forty thousand death certificates recorded over 18 years. Women and men exhibit the same rhythm pattern as the general population, and pneumonia was the primary cause of death in PD, consistent with earlier studies. Reduced sunlight intake is a long-term exposure that raises mortality risk directly. On the other hand, low temperatures mediate the higher risk of death in the short term since the low incidence of sunshine produces them. Still, they do not cause a low incidence of sunlight, making them a mediator rather than a confounder. This demonstration is essential because, in the authors’ understanding, it prioritizes the incidence of sunshine, the seasons of the year, and the circadian rhythm at the expense of just a mediator: low temperatures. This offers us optimism that future research will focus on the rhythmicity of Parkinson's disease, potentially leading to novel medicines and reducing deaths.

## Conflicts of interest

The authors declare no conflict of interest.

## Appendix Supplementary materials

Image, application 1
