# Supplementary material for: Seasonality as a risk factor for deaths in Parkinson's disease
Source: Clinics (Sao Paulo). 2024 Oct 25;79:100506. doi: 10.1016/j.clinsp.2024.100506 (PMC11543644; doi:10.1016/j.clinsp.2024.100506)
Supplement: Supplementary file 1 [file mmc1.doc]

CLINICS-D-24-00294

**Supplementary Material**

**Supplemental Material 1** – Fourier transform, annual peak.


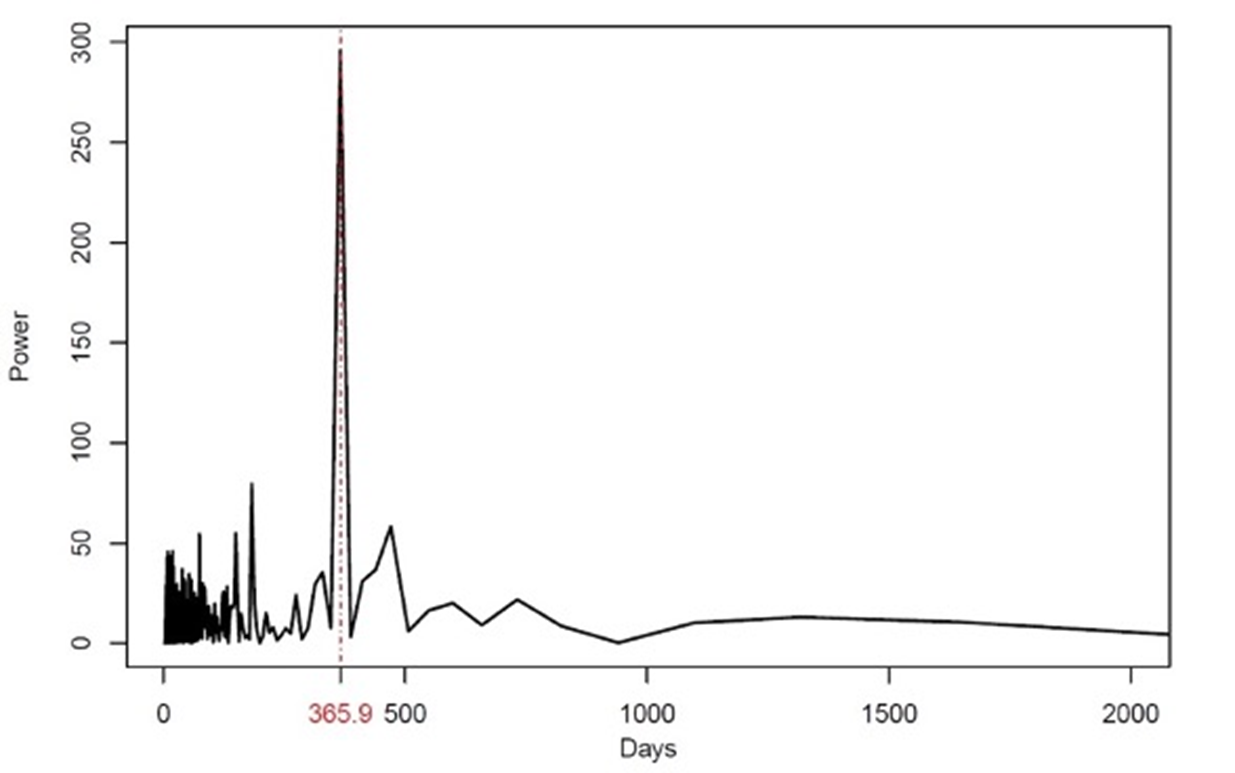


**Supplemental Material 2** – Deaths over the months.


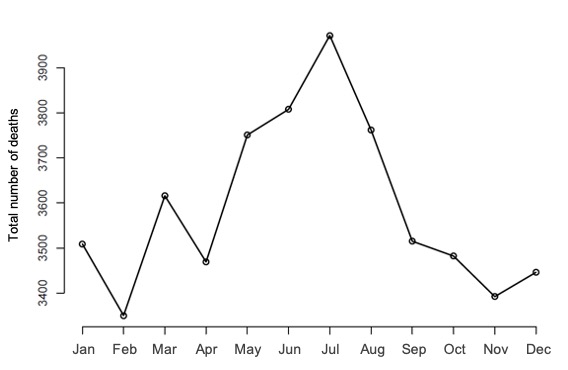


**Supplemental Material 3** – Fourier transform, daily peak.


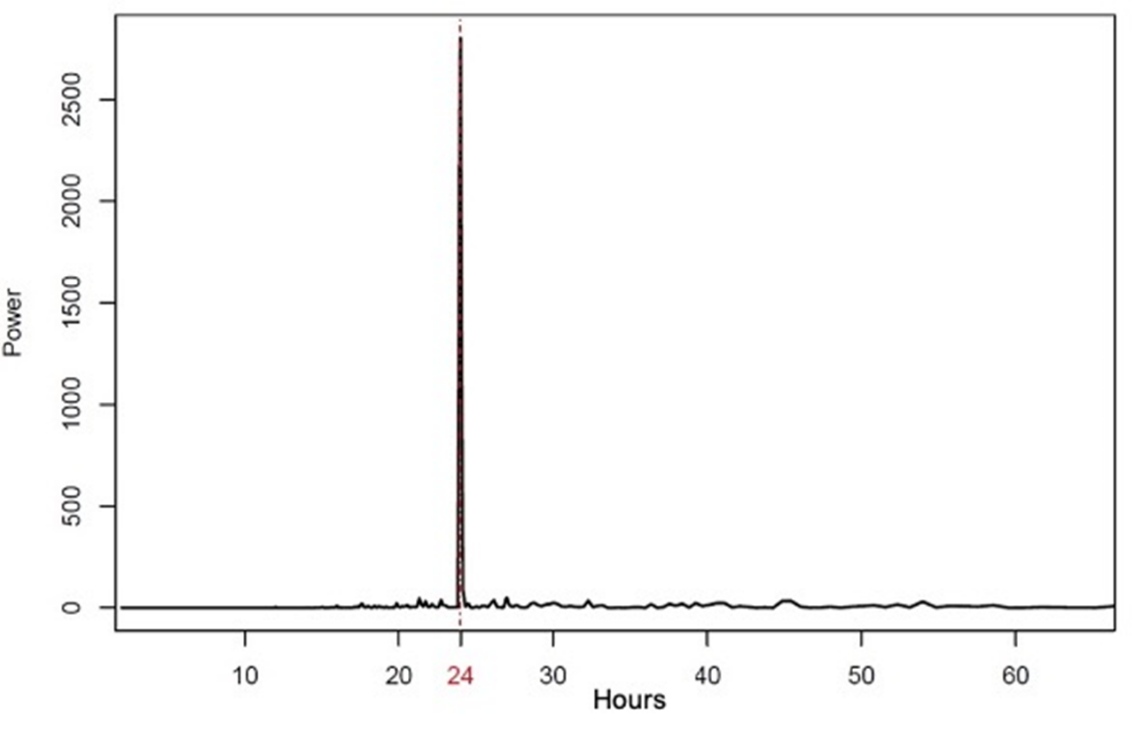


**Supplemental Material 4** – Current hypothesis: the total effect of exposure on the outcome.


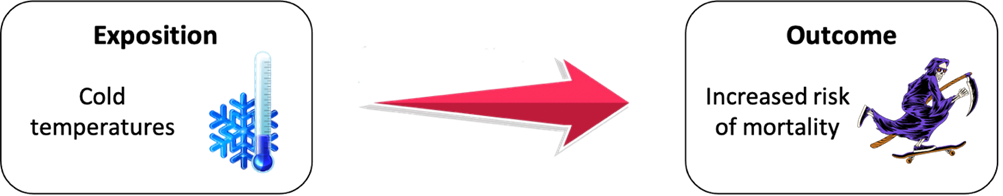


**Supplemental Material 5** – The mediator hypothesis.


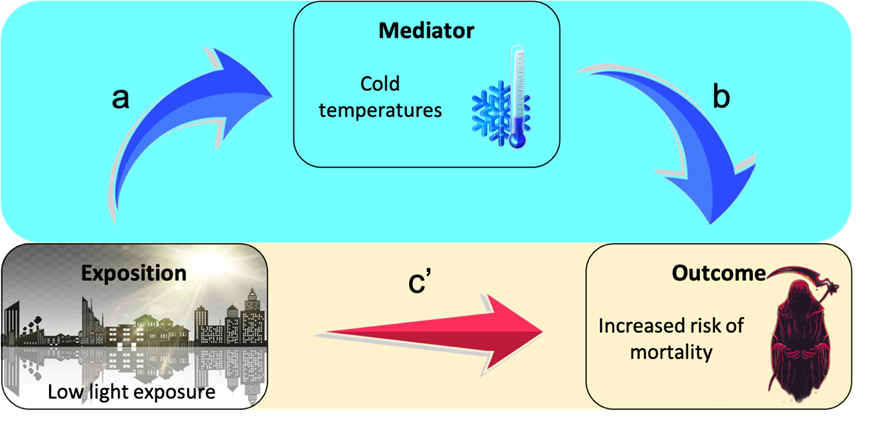


*Legend: Low temperature does not cause low light exposure and is not in the causal pathway between light exposure and increased mortality risk (yellow background: direct pathway); thus, it is not a confounder. Nonetheless, low light exposure can lead to low temperatures. As a result, low temperature is in the causal pathway between low light exposure and increased mortality risk; thus, low temperature mediates some of the impact of low light exposure (partial mediation) (light blue background: indirect pathway).*
